# On the Treatment of Phagedenic Gangrenous Venereal Sores

**Published:** 1875-10

**Authors:** D. B. Simmons


On the Treatment of Phagedenic Gangrenous Venereal Sores. *By D. B. Simmons, M. D.*—Within the last two years several cases which will admit of this classification have come into the hospital for treatment. The hot iron, nitric, chromic, and carbolic acids were all tried in turn, as well as "Ricord's born enemy of phagedenic," with what we believe to be the average rate of success.

The last four cases, and one especially, which we shall refer to, were almost entirely treated, however, by, if not a new process, one which was productive of such satisfactory results as to warrant us earnestly recommending its trial. *This consists in the continuous immersion of the deceased part in hot or warm water.*

. . . It is our opinion that the destructive agency is to be found in the peculiar or specific character of the discharge, and that the water simply removes or dilutes it, so as to destroy its action, the same as it would with a caustic.—*Medical Record.*

